# Relationship between patient ethnicity and prevalence of anemia during pregnancy and the puerperium period and compliance with healthcare recommendations - implications for targeted health policy

**DOI:** 10.1186/s13584-020-00423-z

**Published:** 2020-11-30

**Authors:** Enav Yefet, Avishag Yossef, Manal Massalha, Abeer Suleiman, Aliza Hatokay, Moria Kamhine-Yefet, Zohar Nachum

**Affiliations:** 1 Department of Obstetrics & Gynecology, Emek Medical Center, Afula, Israel; 2 Department of Obstetrics & Gynecology, Baruch Padeh Medical Center Poriya, Tiberias, Israel; 3 Azrieli Faculty of Medicine, Bar-Ilan University, Safed, Israel; 4 Department of Obstetrics & Gynecology, Holy Family Hospital, Nazareth, Israel; 5 Mental health clinic, Clalit Health Services, Yokneam, Israel; 6 Rappaport Faculty of Medicine, Technion, Haifa, Israel

**Keywords:** Ethnicity, Anemia, Pregnancy, Postpartum, Healthcare recommendations, Quality of life

## Abstract

**Background:**

Anemia is common during pregnancy and the puerperium. The association of ethnicity as well as other characteristics with anemia and compliance with healthcare recommendations has not been studied sufficiently and needs to be explored in order to implement a targeted health policy. We examined the association between ethnicity and the risk for prenatal and puerperium anemia and the compliance with healthcare recommendations. This effort aims to guide reforms in policies and practices that will assist in decreasing anemia prevalence in Israel.

**Methods:**

This study was a secondary analysis of a prospective cohort study database including 1558 women who delivered vaginally at Emek Medical Center. Anemia was assessed before delivery by obtaining a complete blood count (CBC). After delivery, CBCs were taken in cases of postpartum hemorrhage, symptoms consistent with anemia, prenatal anemia or other clinical indications. The study population was divided according to their ethnicity (Jews and Arabs).

The primary outcomes were anemia before delivery, anemia in the immediate postpartum and 6 weeks postpartum, and compliance with healthcare recommendations, which was defined as the rate of women who performed a routine CBC test 6-weeks-postpartum.

**Results:**

The rates of anemia before delivery and in the puerperium period were similar between Jews and Arabs (before delivery: 88 (11%) versus 98 (14%); 6 weeks postpartum: 55 (21%) vs 68 (28%), respectively;*p* > 0.05). Iron supplementation was high in both groups during pregnancy (~ 90%) and lower during the postpartum for Jews compared to Arabs (72% vs 83%,respectively; *P* < .0001). Only one third of the patients performed a CBC 6-weeks-postpartum regardless of ethnicity.

**Conclusion:**

Overall compliance with health recommendation was high during pregnancy but low postpartum and was reflected in anemia persistence regardless of ethnicity.

Because of the adverse long term impact of anemia on patient’s health, new policies need to be developed to improve patient’s compliance postpartum. A possible strategy is to combine the follow-up of the mother with the one of the newborn in the family health stations (Tipat Halav) and the community clinics similarly to the close follow-up during pregnancy. Additional methods may include active summoning for CBC test and assuring iron supplement consumption.

## Background

Anemia is a central public health concern and, if not diagnosed and managed appropriately, can bring to short- and long-term complications such as fatigue, decreased functional capacity, infections, impaired quality of life, poor cognitive performance, emotional instability, increased risk for postpartum depression, poor lactation and increased mortality [1–5]. In addition, maternal iron deficiency anemia has an important effect on child cognitive and emotional development [6–8].

Healthcare costs of anemic patients are as much as twice those of nonanemic patients with the same comorbid conditions [9]. The postpartum care visit is an important opportunity to follow up women at increased risk for certain conditions such as anemia as well as impaired physical health and emotional problems. However, compliance with postpartum healthcare recommendation is not consistent and healthcare services utilization can be absent in 10–40% of puerperal women, depending on the specific area and population [10–12]. Thus, it is important to identify populations at increased risk for postpartum anemia and low compliance with medical surveillance in order to change policies that will assist in decreasing anemia prevalence in Israel and improve patients’ medical care.

Ethnicity and cultural attributions have been previously shown to impact public health [10, 12]. Yet, its impact on pregnancy and postpartum anemia as well as on patient compliance in the peripheral north of Israel, a low socioeconomic area, has never been studied and may be of significant importance in outlining health service policies and medical management.

In the present study, we assessed the relationship between ethnicity together with other characteristics on the risk for pregnancy and puerperium anemia, and compliance with healthcare recommendations.

## Methods

### Design

This is a secondary analysis of data collected within the framework of a prospective cohort trial in which we assessed the efficacy of a screening protocol for postpartum anemia diagnosis and treatment in the maternity ward [13]. The study was conducted at the university-affiliated Emek Medical Center, Israel, between June 29, 2015 and January 27, 2016 (ClinicalTrials.gov Identifier: NCT02434653, date of registration: 28/04/2015). The study was authorized by the local review board of the Emek Medical Center (EMC 112–14) and was performed in accordance with relevant guidelines and regulations of the review board. Participants provided written informed consent.

Women who intended to or eventually delivered vaginally (spontaneous or by vacuum extraction) were assessed for eligibility at the labor and delivery, maternal fetal medicine, or maternity wards. Inclusion criteria were women above 18 years of age, who delivered vaginally. Women with a known allergy to iron sucrose, women who delivered by cesarean section and women with pre-eclampsia with severe features (in whom regular complete blood count [CBC] tests are performed) were no eligible to participate in the study.

Blood samples for CBC analysis were obtained prior to or immediately after delivery, from all the participants.

Additional postpartum CBC tests were performed depending on when the subject was recruited:

1. In women recruited between June 29–October 10, 2015, additional postpartum CBC tests were performed in cases of overt bleeding or symptoms consistent with anemia or hypovolemia, or in women with pre-delivery severe anemia (hemoglobin (Hb) < 8 g/dL).

2. In women recruited between October 11, 2015–January 27, 2016. After delivery, CBC tests were taken for the same indications as the first group of recruited participants and also in cases of pre-delivery anemia (i.e., Hb < 10.5 g/dL), regardless of symptoms.

CBC tests were performed until Hb became stable (< 1 g/dL decrease between two tests performed at minimal interval of 8 h). CBC tests were performed at shorter intervals according to the physician’s discretion in cases of substantial or active bleeding, severe symptoms consistent with hemorrhage or anemia, or hemodynamic instability. In addition, in both groups, additional CBC tests were performed when clinically indicated (e.g., maternal fever and assessment of pre-eclampsia).

Intravenous iron sucrose (500 mg) was administered in cases of Hb ≤ 9.5 g/dL. Iron sucrose + red blood cells were transfused in cases of Hb < 7 g/dL, regardless of symptoms or in cases of Hb < 8 g/dL with anemia-related symptoms.

### Study groups

In the present study women were divided according to their ethnicity to Jews and Arabs. Women belonged to other ethnicities were excluded.

### Study endpoints

The primary endpoints were anemia before delivery (Hb < 10.5 g/dL before or immediately after delivery), early postpartum period until discharge from the maternity ward (Hb < 10 g/dL) and during week 6 postpartum (Hb < 12 g/dL). Anemia in the early postpartum period was assessed in women with available CBC tests. CBC tests 6 weeks postpartum is a routine recommendation in our department. Additional primary endpoint was the compliance for healthcare recommendations, which was assessed by evaluating the rate of women who performed the routine CBC test around 6 weeks postpartum.

Data regarding health status and quality of life before delivery was collected using questionnaires (in Hebrew and Arabic) that were given after delivery, during admission at the maternity ward. Women were asked to rank on a scale of 0 (least disturbing) to 10 (most disturbing) the following parameters for the week before delivery and after delivery: fatigue, dizziness, palpitations, shortness of breath and pre-syncope (sensation of blurred vision or about to faint). During the analysis of the questionnaires we combined the 5 questions regarding anemia symptoms (fatigue, dizziness, palpitations and shortness of breath) into one parameter after calculating the Cronbach Coefficient Alpha> 0.7, which suggests that those questions are well correlated and can be combined. For the matter of simplicity we called this score “anemia-related symptoms (ARS) score”, although those symptoms are not specific to anemia and might be reported in non-anemic persons as well.

Quality of life was assessed by the 36-item short-form Health Survey (SF-36) in Hebrew [14, 15] and Arabic [16], as well as by the Hebrew version of the fatigue severity scale (FSS) [17]. The Arabic version of the FSS was translated back and forth to ensure accuracy. The 36 items in the SF-36 questionnaire were combined into 8 categories during analysis (elaborated in Table 2), with a high score indicated a more favorable health state. FSS was scored such that a high score indicated a less favorable health state.

Data regarding number of syncope events, breastfeeding, iron supplementation during pregnancy and vegetarianism, were collected. In addition, the degree of functional capacity during the week before delivery was rated from 0 (worse) to 10 (best).

At around 6 weeks postpartum, participants were contacted by phone and asked to respond to the questions regarding anemia symptoms, the degree of functional capacity, the duration of breastfeeding, and iron supplements consumption postpartum. The women were also reminded to go to their community Ob/Gyn for a routine check-up and to perform a CBC.

Data regarding demographic and obstetric characteristics were collected. Socioeconomic status was ranked using the 2015 Socio-Economic index of the local municipalities, which is published by the Israeli Central Bureau of Statistics. The index of 2015 is available at:

URL: https://www.cbs.gov.il/he/mediarelease/doclib/2018/351/24_18_351t1.pdf.

The index is calculated by means of factor analysis, and is standardized so that the mean index value for all the local authorities is zero. The index value is the distance of the local authority from the mean value measured by standard deviation units. This index was not available for very small authorities (*n* = 230 and 111 women in the Jews and Arabs group, respectively).

### Statistical analysis

#### Sample size

Assuming a rate of anemia of 10 and 15% in Jews and Arabs, respectively, at all time points it was tested, the available sample size was sufficient to detect this difference with a power of 84% (5% 2-sided alpha). In addition, the available sample size was sufficient to detect 40 and 50% rates of women with compliance with healthcare recommendations in Jews and Arabs, respectively (5% 2-sided alpha, 97% power). We controlled for background characteristics using multiple logistic regressions and multivariate Analysis of variance (ANOVA). Statistical analyses were carried out using the SAS version 9.4 (SAS Institute, Cary, NC, USA). Significance was set at a *p* value < 0.05.

Data generated from this study are available upon reasonable request and in accordance with the local institutional review board regulations.

### Results

**Fig. 1:**
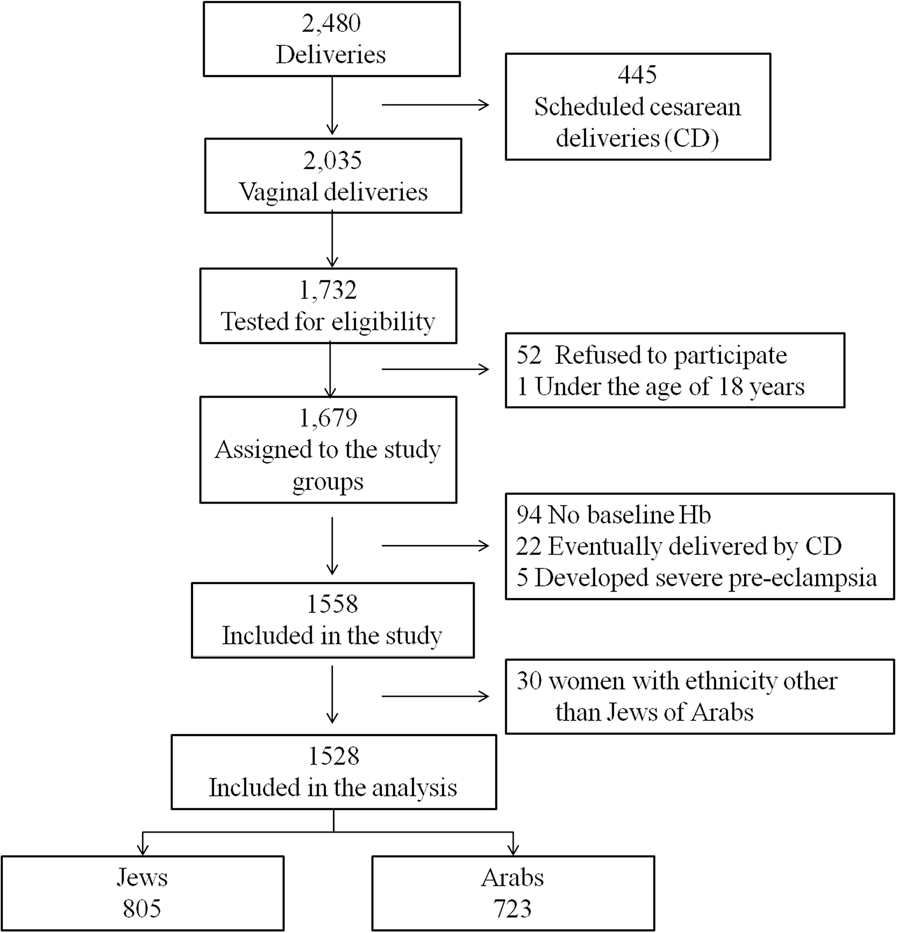
Patients’ flow chart

Figure 1 describes the patients’ flow chart. In total, 1732 women were screened for eligibility, 1679 were recruited and 1588 included in the study. Thirty women with ethnicity other than Jews or Arabs were excluded.

Jewish women were older, but less likely to present with beta thalassemia minor. More Jewish women lived in large cities of higher socioeconomic status and during labor, showed higher rates of epidural analgesia and a lower rate of labor inductions (Table 1).

**Table 1:** Patient demographics and baseline characteristics

Group	Jews	Arabs	*p* value
N	805	723	
Maternal age (years)	30.4 (5.0) [30,27–34]	27.8 (5.2) [27,24–31]	<.0001
Number of deliveries	2.4 (1.5) [2,1-3]	2.4 (1.3) [2,1-3]	0.13
Primiparity	256 (32%)	235 (33%)	0.77
Delivery week	39.4 (1.4) [39.6,38.4–40.4]	39.3 (1.4) [39.4,38.4–40.3]	0.20
Group of original study			
Symptoms	391 (49%)	402 (56%)	0.006
Screening	414 (41%)	321 (44%)	
Beta thalassemia minor	4 (0.5%)	22 (3%)	0.0001
Smoking	7 (0.9%)	1 (0.1%)	0.07
Place of residence			
City > 20,000 residents	441 (55%)	327 (45%)	0.0002
Village ≤20,000 residents	364 (45%)	396 (55%)	
Socio-Economic Index^a^	(−0.1) (0.4) [(− 0.1),(− 0.2)-(− 0.1)]	(− 1.1) (0.4) [(− 1.2),(1.3)-(− 0.8)]	<.0001
Pre-pregnancy BMI	23.4 (4.4) [22.5,20.6–25.5]	24.3 (4.6) [23.6,21.3–26.8]	<.0001
Gestational diabetes mellitus	65 (8%)	74 (10%)	0.14
Pre-gestational diabetesmellitus	7 (0.9%)	5 (0.7%)	0.69
Chronic hypertension	8 (1%)	7 (1%)	0.95
Gestational hypertension	25 (3.1%)	25 (3.5%)	0.7
Epidural analgesia	370 (46%)	182 (25%)	<.0001
Labor induction	261 (32%)	297 (41%)	0.0005
Revision of uterine cavity and cervix (due to hemorrhage)	31 (4%)	28 (4%)	0.98
Manual removal of placenta	11 (1.4%)	8 (1.1%)	0.65
Vacuum extraction delivery	40 (5%)	19 (3%)	0.02
Prolonged second stage of delivery	27 (3.4%)	11 (1.5%)	0.02
Shoulder dystocia	6 (0.8%)	4 (0.6%)	0.76
Perineal tear ≥ grade 3 (involving anal sphincter)	5 (0.6%)	4 (0.6%)	1
Episiotomy	65 (8%)	67 (9%)	0.41

Values are presented as mean (SD) [median, IQR] or number (percent)

^a^ This index was not available for very small authorities (missing to 230 and 111 women in the Jews and Arabs group, respectively)

Pre-pregnancy BMI (body mass index) data were missing for 9 women

Before delivery and during the early postpartum period, anemia rate was similar between women of Arab vs. Jewish ethnicity (Table 2). However, Arab women reported on higher rates of anemia-related symptoms, as manifested by their higher ASR scores (Table 2).

**Table 2:** Study endpoints before delivery, at early postpartum period and 6 weeks postpartum

Group	N	Jews	N	Arabs	*p* value
Before delivery					
Hb	805	12.0 (1.2) [12.0,11.2–12.8]	723	11.9 (1.3) [11.9,11.0–12.7]	0.07
HCT	805	35.1 (3.3) [35.2,33.2–37.3]	723	34.8 (3.5) [34.8,32.5–37.1]	0.03
Hb < 10.5 g/dL	805	88 (11%)	723	98 (14%)	0.11
Hb < 10 g/dL	805	49 (6%)	723	56 (8%)	0.2
Hb ≤ 9.5 g/dL	805	26 (3%)	723	31 (4%)	0.28
ARS score ^ad^	628	1.7 (1.8) [1.2,0.4–2.6]	570	2.6 (2.2) [2.4,0.8–4]	<.0001
Early postpartum (during maternity department admission)					
Hb < 10 g/dL^b^	216	121 (56%)	197	115 (58%)	0.63
Hb < 9.5 g/dL ^b^	216	101 (47%)	197	102 (52%)	0.31
Iron supplementation during pregnancy	572	518 (90%)	493	454 (92%)	0.38
Treatment with IV iron sucrose for postpartum anemia	805	89 (11%)	723	106 (15%)	0.04
Breastfeeding during admission at maternity department	610	533 (87%)	561	539 (96%)	<.0001
SF-36 items^c^					
SF-36 Physical Functioning^d^	602	73.3 (26.5) [80,55–95]	555	70.5 (24.4) [75,55–90]	0.003
SF-36 Role limitations due to physical health	595	32.9 (39.7) [25,0–75]	543	37.5 (41.8) [25,0–75]	0.1
SF-36 Pain	585	53.5 (24.0) [55.0,32.5–67.5]	543	51.7 (24.7) [45.0,35.0–67.5]	0.31
SF-36 General Health^d^	611	75.6 (14.0) [79.2,66.7–87.5]	569	66.3 (15.1) [66.7,58.3–75.0]	<.0001
SF-36 Energy/fatigue	580	48.6 (18.1) [50,35–60]	529	47.4 (21.5) [50,30–60]	0.34
SF-36 Social functioning^d^	591	78.1 (19.7) [80.0,65.0–100]	541	70.6 (24.1) [77.5,55.0–90.0]	<.0001
SF-36 Role limitations due to^d^ emotional problems	585	67.2 (43.0) [100,33.3–100]	539	53.7 (45.4) [66.7,0–100]	<.0001
SF-36 Emotional well-being^d^	580	69.9 (10.6) [72,64–76]	530	61.3 (15.2) [64,52–72]	<.0001
ARS score after delivery^ad^	625	1.9 (2.0) [1.4,0.6–2.8]	570	2.3 (2.2) [1.8,0.6–3.4]	0.006
Fatigue severity scale scores^a^	570	32.8 (12.4) [33,23–42]	521	33.1 (14.1) [33,21–44]	0.12
6 weeks Postpartum					
Breastfeeding ≥30 days	744	474 (64%)	647	539 (83%)	<.0001
Iron supplementation during postpartum period	743	532 (72%)	647	537 (83%)	<.0001
ARS score^ad^	745	0.5 (1.1) [0,0–0.4]	646	0.9 (1.4) [0,0–1.2]	<.0001
Functional capacity^d^	744	9.2 (1.5) [9, 10]	646	8.9 (1.8) [8–10]	0.007
Performed CBC	805	265 (33%)	723	242 (34%)	0.82
Hb (g/dL)	265	12.7 (0.9) [12.7,12.1–13.3]	242	12.4 (1.0) [12.4,11.7–13.1]	<.0001
Hb < 12 (g/dL)	265	55 (21%)	242	68 (28%)	0.054
Hb < 11 (g/dL) ^d^	265	8 (3%)	242	23 (10%)	0.002

Values are presented as mean (SD) [median, IQR] or number (percent)

^a^ Anemia-related symptoms score was comprised of 5 questions regarding symptoms that are related to anemia (fatigue, dizziness, palpitations and shortness of breath), ranked on a scale of 0 (best) to 10 (worst); fatigue severity scale score was scored so that a high score defines a less favorable health state

^b^ Only women that performed CBC postpartum were included

^c^ The SF-36 items were scored such that a high score indicated a more favorable health state

^d^ After adjusting to group of original study, beta thalassemia minor, place of residence, socio-economic index value (below the median value versus at least the median value), only SF-36 Physical Functioning remained statistically significant (p = 0.03). The variable Hb < 11 g/dL was adjusted using multivariate logistic regression. The rest of the variables were adjusted using multivariate ANOVA

*ARS* anemia-related symptoms; *CBC* complete blood count, *Hb* hemoglobin; *HCT* hematocrit

During the early postpartum period, Arab women reported on lower quality of life status as expressed in the lower score for the SF-36 items: physical Functioning, general health, social functioning, role limitations due to emotional problems and emotional well-being, and higher ARS scores as compared to Jewish women (Table 2). Six weeks postpartum, ARS score and functional capacity were less favorable for Arab women. During this period, more Arab then Jewish women breastfed and took iron supplements. Nevertheless, the overall rate of anemia was similar between the groups, although the rate of Hb < 11 g/dL was higher among Arab women. Only one third of the women in each cohort performed the routine recommended CBC test (Table 2).

When adjusted the statistically significant different SF-36 items and ARS scores for group of original study, beta thalassemia minor, place of residence and Socio-Economic Index Value (below the median value versus at least the median value), only SF-36 Physical Functioning item continued to show a statistically significant difference between the two cohorts, with less favorable scores among the Arab women (73.3 ± 26.5 versus 70.5 ± 24.4 for Jews and Arabs, respectively; *P* = 0.03 in multivariable ANOVA).

### Discussion

The present study aimed to explore a potential association between ethnicity and increased risk for peri-delivery- and puerperium anemia, and compliance with healthcare recommendations among women in the peripheral north of Israel. The results suggest that anemia before and immediately after delivery were not significantly different between the two ethnic groups. However, 6 weeks postpartum, the rate of women with Hb < 11 g/dL was significantly higher among Arab as compared to Jewish women, despite their reportedly higher rates of iron supplements use. The higher rate of beta thalassemia minor in this group may have influenced this parameter, as following adjustment for beta thalassemia, the rate of women with Hb < 11 g/dL lost its statistical significance. The reason why beta thalassemia minor did not affect the rate of anemia near delivery may be because iron deficiency and anemia due to increased plasma volume are more significant factors in this period.

An important finding of this study was that the compliance with healthcare recommendations postpartum was both similar and low in the two cohorts, constituting only one third of each group. Iron supplement use was lower postpartum than during pregnancy and to a greater extent in Jews.

Low compliance with healthcare recommendations during the puerperium period has been previously reported and was shown to be impacted by various factors, including social economic status, distance from primary health institutions, and parity, where multiparity increased the likelihood of nonuse or suboptimal performance of postpartum family evaluations in rural areas of eastern China [11]. Similarly, ethnicity affected utilization of healthcare services for postpartum care and was lower among African-Americans compared to non-Hispanic whites in Illinois women with Medicaid-paid deliveries, with respect to receipt of any care, fewer visits, and first postpartum medical visits [10]. In data analysis from 11 states in the United States and New York City, the prevalence of postpartum care visit was lower for mothers with < 8 years of education, mothers who had not received prenatal care, mothers who had received late prenatal care, and mothers whose infants did not have well-baby checkups. The prevalence of postpartum care visits among Hispanic women was lower than for other ethnicities [12]. In southern Israel, Muslim Bedouin women had a higher rate of severe anemia (Hb < 8 g/dL) as compared to Jewish women. Adherence to treatment for anemia was very low in both groups, with overall adherence to treatment reaching only 16, 9.5 and 11.3% for iron, vitamin B12 and folic acid supplements, respectively [18]. In the same study, adherence to iron replacement was higher among Bedouin women compared to Jewish women (17.5% vs. 14.4%, *p* = 0.006), which corroborates the finding in our study, which showed that Arab women used more iron supplements than Jewish women. Similarly, anemia was more prevalent among Arab/Turkish women in spite of receiving more iron prescriptions than Western women in a cross-sectional study conducted in Brussels, Belgium [19]. Ethnicity was shown to affect differently on factors associated with adherence to the use of iron-containing prenatal multivitamin/mineral supplements among low-income pregnant women; among white women, education beyond high school, unmarried status, nulligravidity, and smoking were positively associated with adherence. In contrast, among black women, supplement use 3 mo prior to current pregnancy and no loss of appetite were positively associated with adherence [20].

In the current study, Arab women reported on lower quality of life and higher ARS scores, a finding that is likely unrelated to anemia or healthcare services utilization, as both groups had similar rates of those outcomes. On the contrary, socio-demographic characteristics were probably responsible, at least in part, for these findings, since after controlling for background characteristics, the difference in most of the quality of life parameters and symptoms became statistically insignificant between the groups. These findings suggest that healthcare and welfare services should pay attention not only to ethnicity and cultural attribution but also to socioeconomic characteristics as those may affect the perception of physical health and emotional well being in which healthcare as well as welfare services should intervene.

Low rates of anemia before delivery and in the puerperium and high rates of iron supplementation during pregnancy were documented in both Jews and Arabs. The similar findings in both groups are important as substantial Arab-Jewish differences exist in other medical fields, such as glycemic control of youth with type 1 diabetes mellitus [21] or emergency department utilization [22]. Possible explanations are that with regard to pregnancy, both populations are highly motivated to approach healthcare facilities which are easily accessible.

During pregnancy the follow-up of both mother and fetus takes place at the same time and place, either in the family health stations (Tipat Halav) or the community clinics. After delivery, the follow-up of the newborn becomes separated from the one of the mother except from screening for post-partum depression and breastfeeding that are still addressed during Tipat Halav visit. Based on the findings of this study we suggest combining the follow-up of the mother together with the one of the newborn in Tipat Halav and the community clinics similarly to the close follow-up during pregnancy. This follow-up should take place at the same times of those of the newborn (after 10 days, 1 month and etc.). During those visits the mother will be interviewed regarding iron supplement consumption, screening for postpartum depression, weight gain and loss as well as blood pressure measurement. In addition, in the 1 month postpartum visit she could see the community Ob/Gyn if the physician is present in the same building. Alternatively, she will be reminded to attend the community Ob/Gyn and perform a CBC test and in women with gestational diabetes mellitus 75 g oral glucose tolerance test can be done. Dietician consultation or referral to a dietician can also be done. Additional methods to increase compliance for CBC tests may include active summoning for CBC test by the community clinics by phone, e-mail or text (SMS) message. Future studies to test the usefulness of those suggestions should be conducted.

The strengths of this study include its prospective design, use of validated questionnaires, systematic assessment of symptoms and address of multiple socio-demographic characteristics. The limitations of this study included failure to assess subtypes of anemia and determination of socioeconomic status based on place of residency. In addition, this study was conducted in the north of Israel and the results might not be applicable to other regions and populations such as the Negev Beduins [18].

### Conclusions

The rate of anemia in pregnancy and the postpartum period was not associated with ethnicity in the population of north Israel. Utilization of health services was similarly low between the Jewish and Arab women and national efforts should be made to improve this. Those may include to combine the follow-up of the mother with the one of the newborn in the family health stations (Tipat Halav) and the community clinics similarly to the close follow-up during pregnancy. Additional methods may include active summoning for CBC test and assuring iron supplement consumption. Finally, ethnicity was associated with health symptoms and quality of life parameters, likely rooted in socio-demographic aspects, which should be the focus of national programs as well.

## Data Availability

The datasets used and/or analyzed during the current study are available from the corresponding author on reasonable request.

## Abbreviations

ANOVA: Analysis of variance
ARS: Anemia-related symptoms
BMI: Body mass index
CBC: Complete blood count
FSS: Fatigue severity scale
Hb: Hemoglobin
HCT: Hematocrit
SF-36: The 36-item short-form Health Survey
